# Climate change and international migration: Exploring the macroeconomic channel

**DOI:** 10.1371/journal.pone.0276764

**Published:** 2022-11-16

**Authors:** Albano Rikani, Katja Frieler, Jacob Schewe

**Affiliations:** 1 Potsdam Institute for Climate Impact Research, Potsdam, Germany; 2 Department of Physics and Astronomy, University of Potsdam, Potsdam, Germany; 3 INSERM, Pierre Louis Institute of Epidemiology and Public Health, Sorbonne Université, Paris, France; University of Macerata, ITALY

## Abstract

International migration patterns, at the global level, can to a large extent be explained through economic factors in origin and destination countries. On the other hand, it has been shown that global climate change is likely to affect economic development over the coming decades. Here, we demonstrate how these future climate impacts on national income levels could alter the global migration landscape. Using an empirically calibrated global migration model, we investigate two separate mechanisms. The first is through destination-country income, which has been shown consistently to have a positive effect on immigration. As countries’ income levels relative to each other are projected to change in the future both due to different rates of economic growth and due to different levels of climate change impacts, the relative distribution of immigration across destination countries also changes as a result, all else being equal. Second, emigration rates have been found to have a complex, inverted U-shaped dependence on origin-country income. Given the available migration flow data, it is unclear whether this dependence—found in spatio-temporal panel data—also pertains to changes in a given migration flow over time. If it does, then climate change will additionally affect migration patterns through origin countries’ emigration rates, as the relative and absolute positions of countries on the migration “hump” change. We illustrate these different possibilities, and the corresponding effects of 3°C global warming (above pre-industrial) on global migration patterns, using climate model projections and two different methods for estimating climate change effects on macroeconomic development.

## 1 Introduction

Future global warming is projected to impact both natural and human systems, increasing the climate-related risks to health, food security and economic growth, among others [1]. These climate change impacts may affect human migration flows [2]. Estimating the magnitude and pattern of such phenomenon might be of crucial importance for preparing future societies, and to understand if these responses will act to lower or amplify other climate-related risks, e.g. population’s exposure or vulnerability. However, only few studies have attempted to estimate the effects of climate change on future migration.

Existing projections are mostly limited to extrapolation of past statistical relationships between annual climate variables and aggregate net migration or outmigration for individual countries or regions (see [3] and ref. therein). In such studies, the mechanism that underlies a statistical relationship between migration and, say, annual temperature or rainfall variations often remains unclear. This is problematic because it is difficult to assess whether past relationships can be extrapolated into the future, or whether they will be altered by changes in other, non-environmental drivers of migration [4]. The few examples of projections that explicitly address such mechanisms include migration in Bangladesh due to future sea level rise [5], and Mexico–US migration due to climate-induced changes in crop yields [6]; both again limited to individual countries.

There is thus a research gap related to projections of global migration under climate change based on a mechanistic understanding of the pathways connecting these two processes. Our study aims to contribute to closing this gap by addressing one specific pathway: The indirect effect of climate change on international migration via impacts on macroeconomic development. We provide, for the first time, a set of future projections of climate change impacts on global bilateral migration patterns acting through this macroeconomic channel. In doing so, we explicitly address existing theoretical and empirical uncertainties, by quantifying several alternative assumptions about how macroeconomic changes affect migration dynamics.

In the following section, we first discuss the theoretical and empirical basis for the effect of national income levels on migration, the related uncertainties, and how we reflect them in our study design; before turning to the technical description of data and models used. Section 3 presents the simulation results, and section 4 synthesizes the results and discusses strengths and limitations of our study.

## 2 Materials and methods

### 2.1 Effects of income levels on migration

While there are many other important factors shaping international migration [7, 8], it has been recognised that economic conditions in the origin and the destination country—often measured by gross domestic product (GDP) or similar indicators—are fundamental [9–11]. On the other hand, robust findings in the climate-economics literature suggest that climate change will have significant effects on nations’ economic output, and that these effects will be distributed unevenly across the globe [12, 13]. Thus the question arises: how might such economic impacts of climate change affect global migration patterns?

Addressing this question requires that we account for the economic impacts in both the origin and the destination country, for any bilateral migration flow. Specifically, we must make assumptions about how future income levels, which may be quite different from today’s, will affect international migration. Unfortunately, such assumptions are not easy to constrain with past data, given the short duration of observational migration flow time series, and the high level of short-term variability in observed flows. Indeed, existing migration models cannot predict the observed temporal dynamics in bilateral flows [14]. Besides the short observational period and the incoherent temporal variability of flows, this is also related to the fact that spatial differences tend to be much larger than temporal changes.

Lacking predictive models of changes in migration flows, the approach we take is to construct transparent scenarios of migration under climate change, conditional on certain assumptions about the long-term temporal dynamics of migration. While the temporal signals in observational data are incoherent, there are some clear spatial patterns that emerge from global migration flow data, and it is plausible that some of the mechanisms explaining these spatial patterns also hold valid over time. Specifically, on the side of the destination country, GDP per capita (GDPc) levels have consistently been found to be positively related to immigration [10, 11]; richer countries tend to attract more immigrants. We will assume that this relation, as estimated from historical data, will remain stable in the coming decades; as countries grow relatively richer or poorer, they become relatively more or less attractive, respectively, for migrants, compared to other destinations.

On the side of the origin country, the relation between GDPc and emigration is more complex and ambiguous. Many recent studies have highlighted a non-monotonous, inverted U-shaped, dependence of emigration rates on average income levels in the origin country, which has been called the “migration hump” [15] It has been explained as a combination of migration aspirations that decline with rising incomes, and a number of factors that impede emigration from very poor countries [16, 17]: For instance, international migration is costly, and in poor countries many people may not have the resources to cover these costs even if they would like to emigrate. Thus, emigration tends to be highest in middle-income countries where people can both afford the investment to move, and can still expect a substantial gain in income—or generally, in utility—from moving. Ref. [15] provides a comprehensive overview of this and other factors explaining the migration hump.

It is, however, not fully clear whether the migration hump also reflects a process sometimes termed the migration “transition”: The movement of individual countries along an inverted U-shaped trajectory of emigration rates as incomes gradually rise over the course of decades. Do countries track the migration hump as they grow richer (or poorer)? Available emigration data suggest that they do not, and that emigration rates may instead decline even in poor countries during periods of economic growth [18]. However, the available data is limited in duration and primarily reflects what we consider short-term variations in migration flows, on the order of five years (which is a typical interval of measurement at which global migration data are available). The migration “transition”, on the other hand, is thought to proceed in the long-run as countries grow wealthier in the course of many decades. It is thus currently not possible to determine whether individual countries’ emigration rates do follow the migration hump over time, meanwhile being perturbed by more short-term variability that follows different mechanisms; or whether the migration hump is a purely spatial phenomenon that can be explained solely by differences between countries with regard to other, exogenous factors [18]. This is however a crucial question, because climate change, as a long-term phenomenon, will have gradually growing impacts on countries’ economic development, and thus would affect the rate at which countries progress through the migration transition.

Moreover, supposing that countries do undergo a long-term transition along the migration hump, it is still unclear whether relative or absolute incomes matter for this transition. Some empirical analyses seem to indicate that the peak of the migration hump is associated with higher (real) GDPc values in more recent periods [15, 18]. That is, a certain value of real GDPc would have a country located on the declining branch (to the right of the peak) of the hump in the 1970s, but on the increasing branch (left of the peak) in the 2010s. This may be explained by arguments that as the world as a whole grows richer, it is relative deprivation, rather than absolute poverty, that motivates people to emigrate. However, the empirical evidence for this non-stationarity of the migration hump is weak, and there are also arguments for absolute (real) incomes playing a role both in migration aspirations and in the ability to afford the costs of migration [19].

Given these open questions surrounding one of the most important mechanisms driving migration globally, we test in our study three alternative assumptions about the relation between origin-country GDPc and emigration, using an innovative, non-linear migration model calibrated on historical migration flow data, as described below. The first assumption is that the migration hump is a purely spatial phenomenon, and that countries do not undergo a migration transition as their income levels change on long timescales. To reflect this assumption in our model, we keep emigration rates constant at their average historical levels as we investigate future climate impacts on migration; casually speaking, we “freeze” the migration hump. We refer to this first assumption as CR, for constant rates.

The second, alternative assumption, is that countries undergo a transition along the migration hump with respect to their absolute real income levels; i.e., the migration hump function is stationary in terms of GDPc, and country-level emigration rates change as GDPc levels change. In our model, the migration hump is represented through a simple but effective approximation to a non-parametric regression of the empirical data (see section 2.2.4). We refer to this second assumption as T0 (transition, no change to migration hump function). Finally, the third alternative assumption is that the migration transition occurs with respect to relative income levels. To this end, we shift the migration hump function towards higher GDPc values over time, along with rising global average GDPc. We refer to this third assumption as TS (transition, shifted migration hump function).

We investigate how future climate change affects global migration patterns in our model under each of these three assumptions. Given the lack of well-performing predictive models of migration, as well as the complexity of both migration and climate change and the large number of possible interactions between them, the contribution of our study is to expose a specific, indirect channel of climate impacts on migration; to disentangle it from other potential effects, and to demonstrate how it can be addressed by combining the most recent advances from the climate-economics and migration modeling literature. We do not present predictions of future migration, but a quantification of the effect that climate change may have on migration patterns via a specific channel—the impacts on macroeconomic development –, conditional on the current knowledge and uncertainties about the effects of national income as one of the most important migration drivers.

### 2.2 Data and models

To estimate the potential effect of climate change on future migration, we first derive country-level temperature data from global climate model (GCM) simulations. In order to control for differences in the speed of global warming between different climate models and future greenhouse gas (GHG) concentration scenarios, we focus on a level of 3°C global warming above pre-industrial conditions, rather than on a certain time period. The different climate models are therefore used primarily to represent different possible spatial patterns of warming. In a next step, we calculate the expected effect of climate warming on GDPc, following two alternative methods from the recent climate-economic literature. GDPc projections from the Shared Socio-economic Pathways (SSPs; [20, 21]) serve as a baseline, from which climate change-induced perturbations are calculated. Finally, a global migration model is used to calculate migration flows for both the baseline and the perturbed GDPc scenarios, under different assumptions. The effect of climate change is then obtained by comparing migration patterns between the baseline and the perturbed case.

#### 2.2.1 Data

We use historical data on country-specific total population [22] and migrant stocks [23], defined by country of birth and residence, coming from the UN Department of Economic and Social Affairs. Data on future population growth rate is obtained from the SSP projections dataset [24]. Data on migration flows for calibrating the model is obtained from a recent version of global bilateral migration flows dataset [25]. These flows are reported by country of residence and destination. The data is not disaggregated by country of birth, and therefore does not report transit and return migration flows separately. On the other hand, return and transit migration are included explicitly in our model, following the methods in ref [26].

Data on historical country-level GDPc comes from the Penn World Tables (PWT, [27]) 8.1, reported in terms of 2005 purchasing power parity (PPP), and expanded for including missing countries using the PWT 9.0, after rescaling from 2011 to 2005 PPP [28]. Therefore, these historical values are consistent with the SSP projections data. We use future projections of GDPc under the SSP scenarios produced by the OECD [21]. Data on temperature changes is taken from a set of ten different GCMs (S1 Table in S1 File) available within CMIP6 [29] and bias corrected within the Inter-Sectoral Impact Model Intercomparison Project ISIMIP [30]. Global climate models are mathematical representations of the Earth system components, such as atmosphere and land surface, and of their coupling. These models simulate meteorological variables, such as temperature and precipitation, in response to climate forcing from solar insolation, greenhouse gases, natural and anthropogenic aerosols, and other forcing factors [31]. In this study we use the temperature variation estimated by these climate models. We calculate country-level area-weighted average temperatures starting from gridded data, and construct the temperature change Δ*T* as the difference between two subsequent time steps. For the period 2011–2014 the related temperature data comes from the output of the historical run of each climate model. Finally, the temperature of reference *T*_*i*_(0) in Eq 4 is taken from the observed data of Climate Research Unit (CRU). That is, in all our calculations, absolute temperature data come from observations, and only temperature differences are derived from climate models.

When data was not available for some country, the temperature of the closest country has been used (S1 File). The values of the parameters used for the climate change effect on the GDPc, in Eqs 3 and 4 are taken directly from [13] and reported in Table 1.

**Table 1:** Migration model and climate change effect parameters.

*Variable*	*Parameter*	*Value used*
*Emigration and transit migration*		
Intercept	*a*	0.233 ± 0.004
Diaspora	*α*_*p*_	0.943 ± 0.003
Dest. GDP	*α*_*g*_	0.19 ± 0.01
Orig. GDP	*γ*	-0.0016 ± 0.0004
	$\hat{G}$	$ 35301 ± 9356
	$\tilde{G}$	$ 929 ± 139
*Return migration*		
Intercept	*b*	0.124 ± 0.001
	*R* ^2^	0.69
*Climate change effect on GDP*		
Cross-sectional	*α*_*T*_	-0.023
Panel	*α*_1_	0.00641
	*α*_2_	0.00345
	*β*_1_	-0.00109
	*β*_2_	-0.000718

Estimated values for the global parameters of the migration model, reported with a confidence level of 99% (for *a*, *α*_*p*_, *α*_*g*_) and 66% (for $\gamma ,\tilde{G},\hat{G}$). The used values of the parameters entering the climate change effect methods are reported and come from [13].

#### 2.2.2 Temperature projections

Temperature data are taken from a suite of climate model simulations conducted for the Coupled Model Intercomparison Project (CMIP) phase 6 under historical and future GHG forcing (CMIP6, [29]). Because we are interested in the effects of a substantial global warming of 3°C, we consider two strong GHG forcing scenarios, the SSP3–7.0 and SSP5–8.5, associated with a radiative forcing of approximately 7.0 and 8.5 W/m^2^, respectively, by the end of the century [32]. For each model and scenario, we identify a 30-years period in which the level of 3°C global warming is reached. To this end, we define the global warming level as

$$\begin{array}{c} \Delta T_{s,m}\left(t\right)=\Delta T\left(t_{ref}\right)+{\bar{T}}_{s,m}\left(t\right)-{\bar{T}}_{m}\left(t_{ref}\right). \end{array}$$ (1)

where *s* and *m* define respectively the SSP scenario and the GCM. *t*_*ref*_ is a period of reference corresponding to 1986 − 2005. Δ*T*(*t*_*ref*_) is the observational estimate of global warming in this period relative to pre-industrial conditions, and corresponds to 0.75°C as reported in [33]. ${\bar{T}}_{m}\left(t_{ref}\right)$ is the mean annual global temperature for the period of reference according to the climate model *m*. ${\bar{T}}_{s,m}\left(t\right)$ is the annual global mean temperature averaged for a 30-years period defined as [*t* − 14, *t* + 15], where *t* assumes values on 5-years intervals (i.e. at multiples of 5). The condition Δ*T*_*s*,*m*_(*t*) = 3°C defines the 30-years period of interest. The periods used for each climate model and SSP scenario are reported in the S1 Table in S1 File. It is important to note that, since we are looking at a specific global warming level, the difference between the two GHG scenarios appears only in the rate of warming, i.e. the steepness of the pathway towards the 3°C warming level (more slowly under SSP3–7.0 than under SSP5–8.5). To account for uncertainty in the warming rate as well as in the spatial pattern of warming and internal climate variability, we employ ten different GCMs.

#### 2.2.3 Climate change effect on GDPc

Our baseline projections of future GDPc are taken from the projections produced by the OECD [21] under the five SSP narratives [20]. These narratives employ different assumptions on how our societies will evolve in the future, including changes in economic and political spheres. We focus here on SSP3 and SSP5, which are compatible with our high GHG concentration scenarios while representing rather different assumptions about future economic development. SSP3 results in relatively slow economic growth and a stagnant level of between-country inequality, as measured e.g. by the Gini-index [21]. SSP5, on the other hand, features strong economic growth and at the same time, convergence that strongly reduces between-country inequality. Note that while these two scenarios represent the upper and lower extremes of the SSP scenario space in terms of economic growth, the underlying economic models do not account for short-term shocks that may impede growth, and thus SSP3 may still not be the most pessimistic plausible future scenario [21].

To calculate the effect of warming on GDPc, we combine SSP3 GDPc projections with SSP3–7.0 climate projections, and SSP5 GDPc projections with SSP5–8.5 climate projections. For each of these two baseline scenarios, we calculate two corresponding scenarios of country-level GDPc perturbed by global warming, using two alternative methods of estimating the effect of warming on the economy. The first method, named the long-term case, consists in a cross-sectional analysis of long-run weather averages, which work as proxies for climate variables. This method accounts for any adaptation to climate change that is detectable in the historical data, and assumes that the same climate will have the same effect, despite differences in the geographical location. The second method, labelled short-term case, is based on a panel regression analysis using annual changes of temperature. While this method controls for unobserved heterogeneity, meaning that the same weather will have different effects depending on the location, it does not capture adaptation strategies. The first approach employs the results from the cross-sectional regression model [13], resulting in a log-linear damage function on the GDPc level, as in Eq 10 in ref. [13]:

$$\begin{array}{c} \text{ln}\left(G_{i}^{w}\left(\tau \right)\right)-\text{ln}\left(G_{i}\left(\tau \right)\right)=\alpha_{T}\Delta T_{i}\left(\tau \right)\; \end{array}$$ (2)

where *α*_*T*_ is a negative constant factor. *G*_*i*_(*τ*) is the baseline GDPc level in country *i* at time *τ*, as prescribed by the SSP scenario. $G_{i}^{w}\left(\tau \right)$ is the same but under the climate change impact. The temperature is redefined as the mean of the annual temperature over a 5-years period: $T_{i}\left(\tau \right)=\frac{1}{5}\sum_{l=0}^{4}T_{i}\left(\tau -l\right)$. The total warming in country *i* at time *τ* is defined relative to the temperature of the period of reference (i.e. 2011–2015): Δ*T*_*i*_(*τ*) = *T*_*i*_(*τ*) − *T*_*i*_ (2015). This notation highlights the assumption that the warming happening during a 5-years period affects the GDPc at the end of that same period, and that the impacted GDPc trajectory starts diverging from the baseline after the period of reference. This means that, while considering a period of 3°C level of global warming above pre-industrial conditions, the impact on the GDPc is calculated relative to a recent period, which has already seen ∼ 1°C of warming. Therefore our impact study focuses on the effect of additional 2°C global warming. By solving Eq 2 for $G_{i}^{w}\left(\tau \right)$ we can calculate the projected GDPc under global warming, for a country *i* at time *τ* as in Eq 3:

$$\begin{array}{c} G_{i}^{w}\left(\tau \right)=G_{i}\left(\tau \right)\;\cdot e^{\alpha_{T}\;\Delta T_{i}\left(\tau \right)}. \end{array}$$ (3)

It is worth noting that in case of no further warming, Eq 3 reduces to the GDPc trajectory prescribed by the SSP scenario, otherwise the warming will affect the GDPc once and for all at any time *τ* in the future.

The second approach for estimating the impact of climate change on the economic productivity of the country follows the results from a panel regression model [13]. From equation S3 in the supplementary materials of ref. [13], the warming effect term in the GDPc growth rate can be written as

$$\begin{array}{c} \begin{array}{cc} \delta_{i}\left(t\right)= & \alpha_{1}\Delta T_{i}\left(t\right)\;+\alpha_{2}\Delta T_{i}\left(t-1\right)+\\ & (\beta_{1}\Delta T_{i}\left(t\right)\;+\beta_{2}\Delta T_{i}\left(t-1\right))\cdot \\ & \left(T_{i}\left(0\right)+\sum_{j=1}^{t-1}\Delta T_{i}\left(j\right)\right) \end{array} \end{array}$$ (4)

where *α*_1_, *α*_2_, *β*_1_, *β*_2_ are constant factors. The annual changes in temperature are defined as Δ*T*_*i*_(*t*) = *T*_*i*_(*t*) − *T*_*i*_(*t* − 1), where the *T*_*i*_(*t*) represents the annual temperature in country *i* at time *t*. The temperature of reference *T*_*i*_(0) is the observed temperature in the year 2015 and Δ*T*_*i*_(0) is assumed to be zero. Following the approach for defining the multiplicative damage function in equation S5 of the supplementary materials in ref [13], the GDPc projection under the global warming impact is prescribed by:

$$\begin{array}{c} G_{i}^{w}\left(t\right)=G_{i}\left(t\right)\;\cdot e^{\sum_{l=1}^{t}\delta_{i}\left(l\right)}. \end{array}$$ (5)

That is, in this short-term approach, the history of temperature changes matters, and therefore differences in the rate of warming between climate models or scenarios can affect the results.

#### 2.2.4 Migration model

We use a global model of international migration that is calibrated on historical bilateral flow data [25], and has been shown to represent current patterns of bilateral migration well, despite relying on just a small number of predictor variables [26]. Note that refugee movements are not accounted for in the model, as these are assumed to have different causes and dynamics than regular migration [34].

In our model, population is defined by place of birth and place of residence. This specification allows us to represent three different types of migration: Emigration from the place of birth, transit migration between countries different from the place of birth, and return migration to the country of birth. The model also accounts for the important role of the existing migrant networks (diaspora) at the destination country. The role of diasporas, put simply, is that they facilitate further immigration from the same country of birth; for instance, because of family reunification policies, and because diasporas can provide information and support to new immigrants that help them find employment, housing or financial security [35]. The magnitude of this amplifying effect of diasporas may vary over time in ways hardly predictable, as e.g. countries may change their policies; for our study, we assume that the effect remains at its recent level measured in observed migrant stock and flow data. A third important feature of the model is that it represents the “migration hump”, i.e. the inverse U-shaped dependence of emigration on origin country incomes. Finally, pull factors at the destination country are accounted for by relative average income levels, such that the proportion of immigrants a country receives is related to how attractive it is compared to other countries.

Specifically, the number of moves per 5-year period by migrants of place of birth *k* from residence country *i* to destination country *j*, *M*_*k*,*i*→*j*_, is modeled as:

$$\begin{array}{ccc} M_{k,i\to j}=a_{j}\cdot F\left(G_{i}\right)g_{j}^{\alpha_{g}}\;p_{k,j}^{\alpha_{p}}\;P_{k,i} & \text{for}\;k\neq j, & \text{(6a)}\\ M_{j,i\to j}=b_{i}\cdot P_{j,i}. & & \text{(6b)} \end{array}$$

where Eq 6a describes emigration from place of birth (for *i* = *k*) as well as transit migration (for *i* ≠ *k*), and Eq 6b describes return migration (*k* = *j*). *G*_*i*_ is the GDPc at the origin country *i*, and *g*_*j*_ = *G*_*j*_/*G*_*glob*_ is the GDPc at the destination *j*, relative to the global mean GDPc, *G*_*glob*_. *a*_*j*_ and *b*_*i*_ are country-specific scaling factors which are meant to capture any unobserved factors (i.e. variables for which we do not have measurements to explicitly enter our model) that are country-specific and assumed constant over time. An important example are immigration policies. While policies in the destination affect immigration of foreign nationals [36], we assume that return migration rates are rather influenced by policies in the host country (e.g. the possibility to re-enter the country, [37]) than by those in the country of birth; thus the scaling factor is specific to the country of destination, for emigration from country of birth and transit migration, but specific to the country of residence for return migration.

*P*_*k*,*i*_ is the population of place of birth *k* living in country *i*. It follows that the relative diaspora born in *k* and living in country *j* is defined as $p_{k,j}=\frac{P_{k,j}}{\sum_{i}P_{k,i}}$. Since the SSP scenarios provide only numbers of future total population but we need projections also for the migrant stocks, we use SSP-based population growth rates to construct projections of population stocks defined by country of birth and residence. We construct these projections by starting from the observed population distribution in 2015 and using the recursive Eq 7.

$$\begin{array}{c} P_{i,j}\left(t\right)=P_{i,j}\left(t-s\right)\cdot {\left(1+\rho_{i}\right)}^{s} \end{array}$$ (7)

*P*_*i*,*j*_(*t* − *s*) is the population born in *i* and living in *j* at time *t* − *s*, while *ρ*_*i*_ is the SSP-based annual growth rate for country *i*. *s* is the time step of the model, corresponding to 5 years.

*F*(*G*_*i*_) models the non-linear dependence of emigration rates on the GDPc of the country of origin and is meant to capture the relation between both, resources and desire to migrate, and emigration rates (Fig 2, solid line).

$$\begin{array}{c} F\left(G_{i}\right)=\frac{1}{1+\frac{G_{i}}{\hat{G}}}\cdot \frac{1}{1+e^{-\gamma (G_{i}-\tilde{G})}}. \end{array}$$ (8)

The first term, depending on $\hat{G}$, is assumed to capture the desire to migrate, which reaches a maximum for *G*_*i*_ = 0 and approaches zero for large values of *G*_*i*_. The second term, depending on $\tilde{G}$, is assumed to capture the dependence of the emigration rate on economic resources at the origin: it assumes a minimum for *G*_*i*_ = 0 and approaches the maximum of one for $G_{i}\gg \tilde{G}$.

As we introduced at the beginnng of the Methods section, we test three alternative assumptions about the relation between origin-country GDPc and emigration rates.

The CR, constant rates, assumption is implemented in our model by keeping emigration rates constant at the value prescribed by Eq 8 using the country-level mean GDPc for the historical period (1990–2015). Under the T0 assumption countries undergo a transition along the migration hump with respect to their absolute real income levels.

That is, we evaluate Eq 8 at the historical values of GDPc.

Finally, under the TS assumption the migration transition occurs with respect to relative income levels. To this end, we shift the migration hump function towards higher GDPc values over time, along with rising global average GDPc. The shift depends on the growth rate *r* between the global mean GDPc for the historical period, ${\bar{G}}_{hist}$, and the period of interest ${\bar{G}}_{proj}$:

$$\begin{array}{c} r=\frac{{\bar{G}}_{hist}}{{\bar{G}}_{proj}}-1. \end{array}$$ (9)

The transformation *G*_*i*_ → *G*_*i*_ ⋅ (1 + *r*), with *r* < 0, produces a shift of the migration hump towards the higher values of GDPc (Fig 2, dashed line) and the shifted migration-hump function can be written as follows:

$$\begin{array}{c} F\left(G_{i}\right)=\frac{1}{1+\frac{G_{i}\cdot \left(1+r\right)}{\hat{G}}}\cdot \frac{1}{1+e^{-\gamma (G_{i}\cdot \left(1+r\right)-\tilde{G})}}. \end{array}$$ (10)

While the rate of change *r* is equal for all GDPc points, the actual shift depends on the specific GDPc value and is equal to *G*_*i*_⋅*r*. This means that, on a linear scale, the function will appear as a combination of dilation and translation. If an individual country’s GDPc grows at the same rate as the global average GDPc, then the country’s emigration rate remains constant.

We then produce global bilateral migration flows using a 5-years step and covering the 30-years period defined above. We calculate bilateral migration flows for each country of birth–country of origin–country of destination triad. We calculate this set of global bilateral flows for the baseline case as well as for the case of perturbed GDPc under global warming, for each impact method, GCM and SSP scenario. These computations are repeated under the three assumptions regarding emigration rates (CR,T0,TS). In principle, the baseline population distribution entering our calculations will be different for each climate model and scenario, reflecting the distribution of each 30-years period. For simplicity, we use the same baseline population distribution for all the simulations. Across climate models and SSP scenarios, the average period for reaching the 3°C warming is [2046–2075]. We use the population in this period for all simulations. This means that we break the direct association between socio-economic development and GHG forcing implied in the SSP scenarios. This is not a problem for our study because we are not aiming to produce transient, combined projections of climate, migration, population, and GDP; but rather, to isolate the effect of climate change on migration through GDP while keeping other variables (including initial population) fixed.

We construct our analysis by comparing, separately for each of the assumptions regarding emigration rates and each scenario, the migration flows under climate change impact to the baseline case, after averaging the migration flows on both dimensions of time and climate models.

### 2.3 Model estimation

We estimate the migration model defined in Eq 6a and 6b in three different steps. First we estimate the parameters that shape the emigration rate through the term *F*(*G*_*i*_) on the right-hand side of Eq 6a. To this end we follow the method used in ref [26]. That is, where possible, we exclude return and refugee migration flows from the reported bilateral flows. We then aggregate the flows for each country of origin and divide the total outflow by the number of total population living in the country, to obtain emigration rates. Ultimately, we fit, through a nonlinear least square (NLS) method, the emigration rate to the right-hand side of Eq 8:

$$\begin{array}{c} \frac{1}{P_{i}}\sum_{l}{\tilde{M}}_{,i\to l}\approx a_{e}\cdot \frac{1}{1+\frac{G_{i}}{\hat{G}}}\cdot \frac{1}{1+e^{-\gamma (G_{i}-\tilde{G})}} \end{array}$$ (11)

where *a*_*e*_ is a scaling factor. In the second step we fit international bilateral migration flows, that is the count of international population movements, against economic (e.g. GDP per capita) and demographic (e.g. relative diaspora stocks) factors. We split the country specific scaling factors *a*_*j*_ and *b*_*i*_ into the product of a global factor *a* and *b* and country-specific factors ${\tilde{a}}_{j}$ and ${\tilde{b}}_{i}$, respectively: $a_{i}=a\cdot {\tilde{a}}_{j}$ and $b_{i}=b\cdot {\tilde{b}}_{i}$. While the migration data reports only aggregated origin to destination flows, our model would distinguish between the three different channels (transit, return and emigration from country of birth) contributing to the total bilateral flow. Reproducing the approach in ref. [26] we fit the reported [25] bilateral migration flows *M*_*i*,*j*_ to the sum of emigration from country of birth and return migration flows defined in our model in Eq 6a and 6b:

$$\begin{array}{c} M_{i\to j}^{obs}\approx M_{i,i\to j}+M_{j,i\to j}. \end{array}$$ (12)

Due to computational costs, we do not include the transit flows. These flows have been shown to have a very small share in the total emigration compared to return and emigration from country of birth flows [38]. While presenting its limitations this method is still an improvement in the granularity of the modeled flows compared to previous methods. The parameters entering in *F*(*G*_*i*_) are set to the values estimated in the first step, while the country-specific scaling factors ${\tilde{a}}_{j}$ and ${\tilde{b}}_{i}$ are not included here. We use a (NLS) method of estimation which allows us to implement the additive form of the model. Commonly, previous studies log-transform the model in order to interpret the coefficients as elasticities. The additive form of our model does not allow us to follow this procedure. Nonetheless it produces results in line with previous works, with the diaspora representing a strong pulling factor and the destination GDPc being positively correlated to the migration flow. In the last step, using an OLS method, we estimate the country-specific scaling factors on the mean bilateral migration flows, while keeping the remaining parameters set at the values estimated in the previous steps.

Gravity models are often estimated through a Pseudo-Poisson maximum likelihood (PPML) estimator, reducing the risk of obtaining biased estimates, or by a log-log transformation which allows to interpret the coefficients as elasticities using OLS methods. Our model specification refrains us from using any of these methods. The log-log transformation would reduce the data by dropping the zero migration flows as well as those flows that have a zero diaspora value. This could itself lead to strongly biased estimates. The additive form of our model, where we fit the bilateral migration flow as the sum of return and emigration from country of birth flow, makes the log-log transformation obsolete. The same considerations hold for using the PPML method. Finally, alternative specifications of the model where dyadic variables as common border, official language and colonial ties as well as the geographical distance are tested in our analysis. Our estimates of these variables are in many cases statistically not significant or inconsistent. This is likely because inclusion of the diaspora variable already captures the effects of other dyadic variables to a large extent. At the same time, our parameter estimates are largely very robust against inclusion of additional dyadic variables. We report these results in the S3 Table in S1 File. It is important to point out that our model deliberately breaks with common gravity model specifications in order to be able to include the non-linear effect of origin income levels and to account for return migration and transit migration. These innovations are central for the analysis presented in this paper.

The estimated global parameters for the migration model and the values of the parameters used for the climate change effect are reported in Table 1. The value of the country specific scaling factors are reported in the S2 Table in S1 File.

## 3 Results

**Fig 1:**
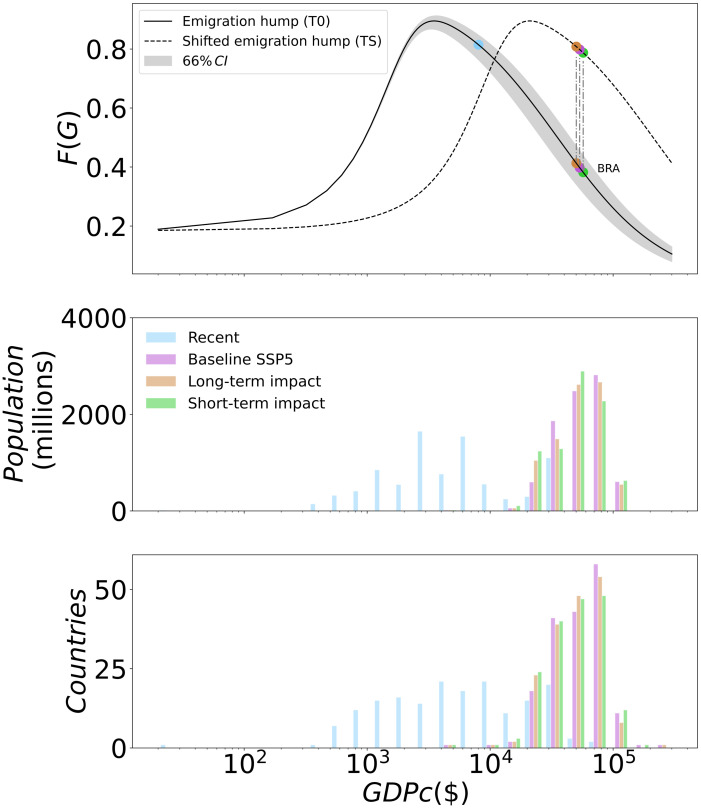
Country level, climate change impact on GDPc under SSP5–8.5 scenario. Positive values represent gains or increase in GDPc under the climate change impact case. The change is calculated as relative to a baseline scenario without climate change impact. Values represent the mean reached within the 30-years period of 3°C global warming (see Methods). Time averaged values are then averaged along the climate models ensemble. Panels (a) and (b) show the impact for the long-term and short-term impact method respectively.

We estimate the impact of future global warming on global migration patterns via the indirect channel of national GDPc. We employ two methods of climate change impact on GDPc, producing two different patterns of GDPc changes due to global warming (Fig 1 and S2 Fig in S1 File).

**Fig 2:**
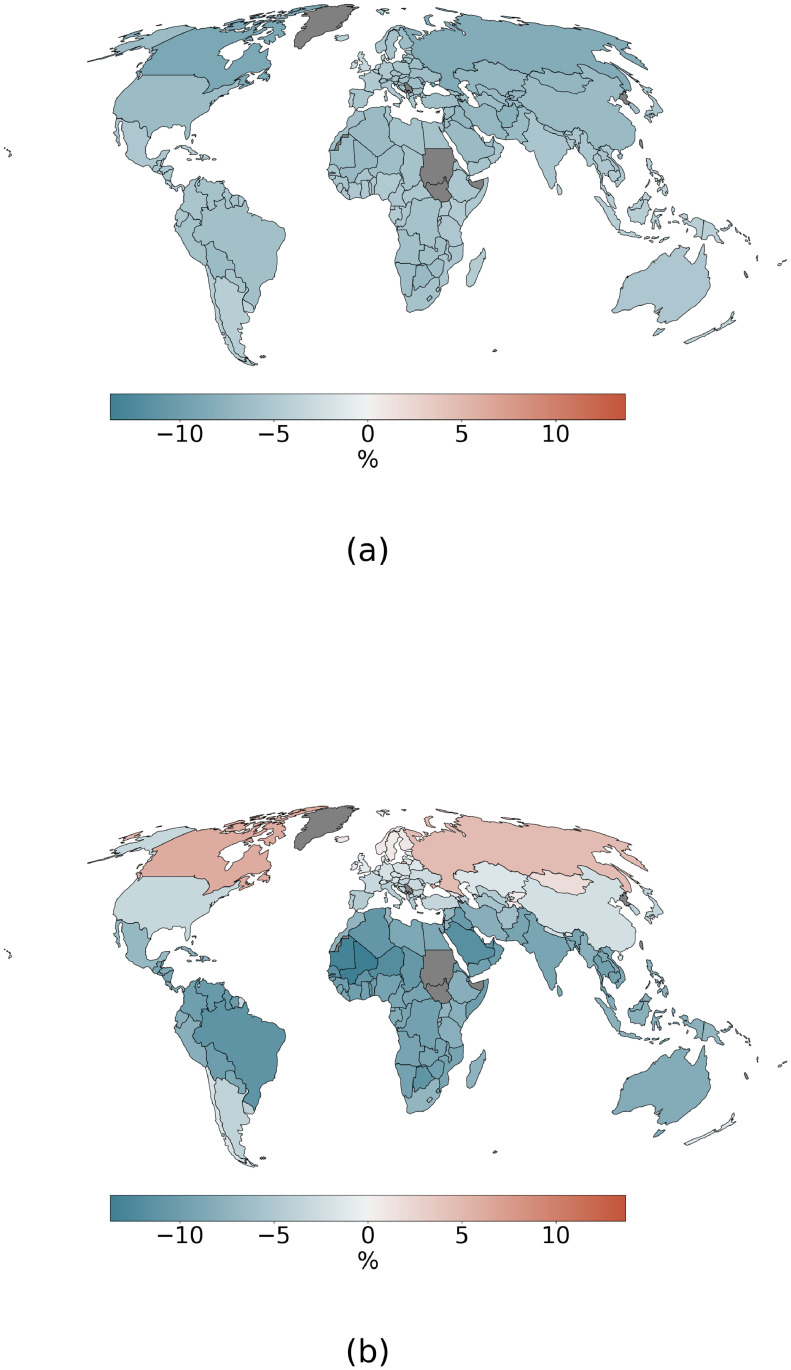
Migration-hump function and GDPc distribution for SSP5–8.5. The upper panel shows the emigration hump function for the T0 assumption (black line), as described in Eq 8, and its shifted version for the TS assumption (dashed line), as described by Eq 10. We show for one country its location on both these curves and its location when considering the CR assumption (blue dot). Grey area represents the extremes reached by the migration hump function when considering the parameter $\hat{G}$ on its values at 66% confidence interval. The middle panel shows the population weighted GDPc distribution, for the baseline SSP5, for both climate change impact methods and for the CR assumption. Population and GDPc are averaged for the 30-years period of 3°C global warming and over the climate models dimension. The bottom panel shows the same as the middle panel but for the number of countries instead of the population. When considering the constant emigration rate case (blue) we use the mean GDPc distribution for the historical period (1990–2015). We calculate bilateral migration flows using baseline and impacted GDPc trajectories under different scenarios, climate models and emigration rates assumptions. Flows cover a 30-years period (see Methods) and are then averaged on both, time and climate models dimension. For each scenario and emigration rate assumption we compare averaged migration flows produced using the impacted GDPc trajectory to those using the baseline GDPc.

The baseline and perturbed trajectories of GDPc assume different patterns in terms of country- and population-weighted distributions, producing different responses in terms of the assumptions regarding the migration-hump function (Fig 2).

**Fig 3:**
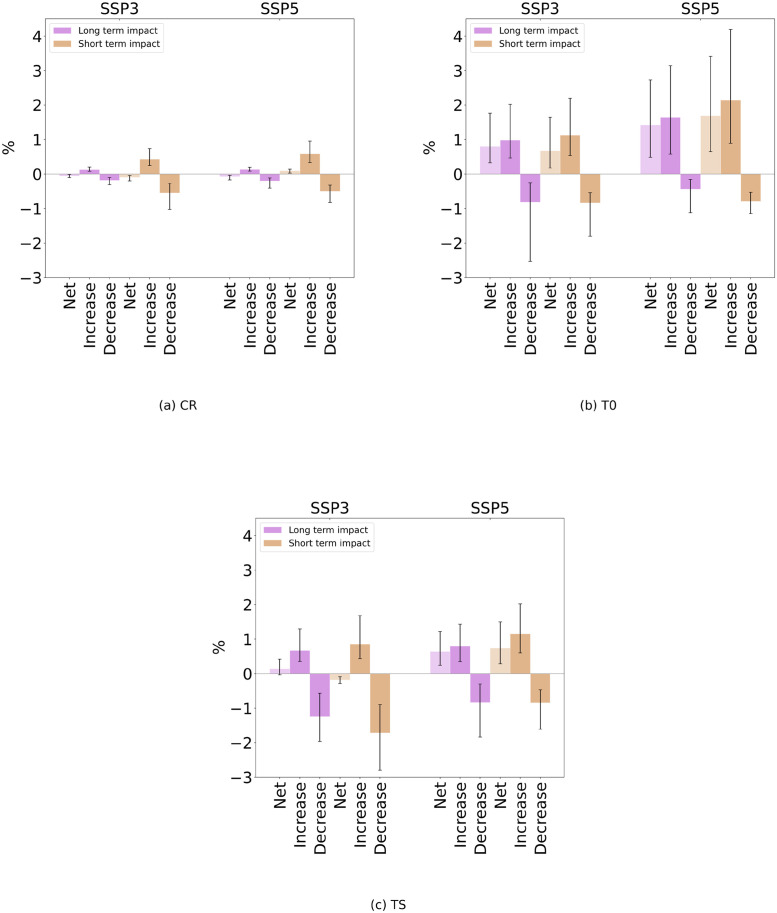
Mean global migration change due to the climate change impact, for each SSP scenario, climate change impact method and emigration rates assumption. The change, in percentage, is computed as the difference of migration under the climate change impact scenario and the baseline case without climate change impact, divided by the baseline case. Positive values define an increase under climate change impact. Flows are averaged over the period of 30 years where the 3°C global warming level is reached (see Methods). For each case we show separately the total (net) change of migration, the change in the total flows that increased and in those that decreased. The error bars represent the extremes reached within the ensemble of GCMs that we use, while the bars show the mean value reached within the set of GCMs. Each panel refers to one assumption regarding emigration rates.

At the global level, under the CR case (constant emigration rates) we find that net change in global migration, i.e. the change in the total number of movements per 5-year period, on average, reaches a maximum of ∼ 0.1% (Fig 3a, light-colored bars). For almost all combinations of SSP scenario and impact method migration is projected to be smaller under climate change impact compared to the baseline. While the figure of the net difference in migration is informative for the overall change in global migration, it does not provide full insight into the magnitude of the impact of climate change on migration flows, because climate change may reinforce some migration channels but inhibit others. Therefore, a more comprehensive picture can be provided by looking separately at the total change in increased and decreased flows. We construct these figures by identifying all bilateral flows that are projected to decrease and all those that are projected to increase. We sum up separately these two set of flows, for both the baseline and climate change scenario, and then we show by how many percent these total number changes. Summing up these quantities we can define the migration movements that would be affected, either inhibited or reinforced. Under all the SSP scenarios and impact methods, the number of increases (i.e. additional moves due to climate change) is similar to the number of decreases (i.e. fewer moves due to climate change), summing up to a maximum of ∼ 1.1% of total affected movements (Fig 3a, dark-colored bars). Positive and larger impact is found in the net change of migration when considering the T0 case (migration transition, no change to migration hump function) (Fig 3b). The total number of affected migration movements remains substantially larger than the net change, reaching a peak of ∼ 3% (increases of about 2%, decreases of about 1%) under SSP5–8.5, short-term method. When considering the TS case (migration transition, shifted migration hump) the impact on the net change remains small under SSP3–7.0 but increases substantially under SSP5–8.5 (Fig 3c). As for the CR and T0 case, the total movements affected by climate change is larger than the net change, in many cases being larger than 2-folds the value of the net change. Altogether these results can be summarized as follows: SSP5–8.5 produces larger differences in migration than SSP3–7.0, short-term impact method leads to larger effects compared to the long-term method and the TS produces lower differences compared to the T0 case. The climate change-induced difference in migration flows is smaller under CR case than under both T0 and TS assumptions.

These results are largely understood by referring to the functional form assumed for the emigration rates (Eqs 8 and 10), destination country GDPc term (Eq 6a) and for the climate change impact methods (Eqs 3 and 5). Under the CR assumption the only difference between the climate change case and the baseline is in the destination country GDPc value in Eq 6a. From our results it follows that this factor alone has only a limited effect on the number of migration moves globally; which is expected from the design of the model, in which destination GDPc mainly controls the distribution, rather than the total number, of migrants.

Turning to the two cases where climate change additionally affects emigration rates through changing origin-country GDPc, the effect appears smaller under the TS assumption compared to the T0 assumption. This is explained by comparing the relation between emigration and origin GDPc (the migration hump function, upper panel in Fig 2) in each case to the future GDPc distribution among countries and population (lower panels in Fig 2). By the time 3°C global warming are reached, most countries are projected to have grown rich enough that they are located to the right of the current peak of the migration hump, i.e. on the declining branch of the unchanged hump function (T0). That means that for many countries, the emigration-GDPc relation is relatively steep, and small changes in GDPc (induced by climate change) lead to relatively large changes in emigration. In contrast, when the hump function is shifted along with global growth in GDPc (TS), more countries are still close to its peak, meaning a flatter emigration-GDPc relation and, thus, relatively small changes in emigration in response to climate change-induced GDPc changes. In addition, for the same reason, more countries will experience a decrease in emigration due to climate change (being located to the left of the peak) under the TS assumption than under the T0 assumption.

Comparing the two different methods of estimating the economic impacts of climate change, the short-term method produces larger and more divergent economic effects compared to the long-term method, with countries in northern latitudes experiencing smaller losses or even benefits [13] (Fig 1). This translates, in terms of relative GDPc, into larger between-country inequalities, which, under the CR assumption, reinforce migration to those high-income countries that see a larger gain in GDPc compared to that experienced by global mean GDPc. All else being equal the short-term impact method produces in many countries larger losses than the long-term case. Larger losses imply that these countries are pushed towards lower values of GDPc under the short-term method, resulting in larger emigration rates for the countries that have already crossed the peak of the emigration function (Fig 2 and S1 Fig in S1 File).

We now discuss changes in the spatial patterns of migration flows, focusing for clarity on SSP5–8.5 and the short-term impact method; corresponding results for SSP3–7.0 and the long-term impact method are displayed in the S1 File.

**Fig 4:**
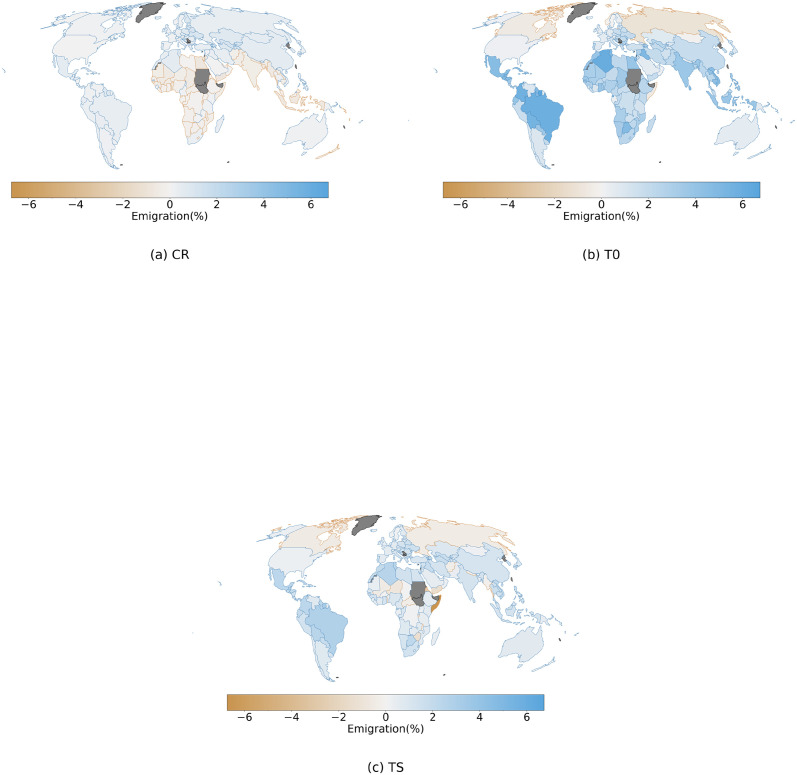
Country level, climate change impact on emigration under SSP5–8.5 scenario and short-term impact method for the three different assumptions regarding emigration rates. Positive values represent increase in emigration flows under the climate change impact case. The change is calculated as relative to a baseline scenario without climate change impact. Values represent the mean reached within the 30-years period of 3°C global warming (see Methods), and are then averaged along the ensemble of GCMs. Panel (a) shows the change under the CR assumption, panel (b) covers the T0 assumption and panel (c) the TS assumption.

At the country level, mirroring the results on the global level, we find that the impact on total emigration is much smaller under the CR assumption compared to the T0 and TS assumptions (Fig 4a). Under CR, an increase in emigration is projected in most regions of the world, except for Africa and Southeast Asia, where climate change is expected to decrease emigration for almost all the countries in the region. Since under this assumption emigration rates are held constant, the effect is attributable to only the change in destination country relative GDPc: the GDPc in the main destination countries for the African and Southeast Asian region is reduced more than the global mean GDPc. Importantly, this divergent effect is driven by the uneven distribution of economic impacts under the short-term impact method, where many large destination countries at high northern latitudes experience only small losses or even gains from climate change.

When we assume that emigration rates respond to changes in origin GDPc according to the original migration hump function (assumption T0), we find a very different pattern than under assumption CR. Emigration is projected to increase for most countries, and most strongly so in many tropical and subtropical countries (Fig 4b). Decreases in emigration are found in high northern latitude countries. In other words, emigration is projected to increase from countries that experience economic damages; while it would decrease from countries that gain from the impacts of climate change. Again, this pattern can be explained through the emigration function implemented in our study. Indeed, under the baseline SSP 5 scenario, all countries have already crossed the peak of the migration-hump function and a negative impact would move them towards lower levels of GDPc, associated with higher rates of emigration. The opposite applies to countries like Canada, which are projected to experience a positive impact on the GDPc.

The same process drives the results from the TS assumption, where results are similar as under the T0 assumption. However, since the GDPc distribution of the countries covers a portion of the emigration function closer to the peak, we find two main differences with the T0 assumption: Different countries in Africa and Southeast Asia show a decrease in emigration, and the range of values reached is smaller than under the T0 assumption (Fig 4c). The shifted emigration function in this case redefines the position of the peak, mapping a portion of the countries on its left branch, where a loss in GDPc turns into a decrease in emigration. While few countries may find themselves on the left branch of the new shifted migration-hump, many countries get closer to the peak, where rates of change in emigration rates are lower and therefore the same impact on the GDPc would produce smaller changes under the TS assumption.

**Fig 5:**
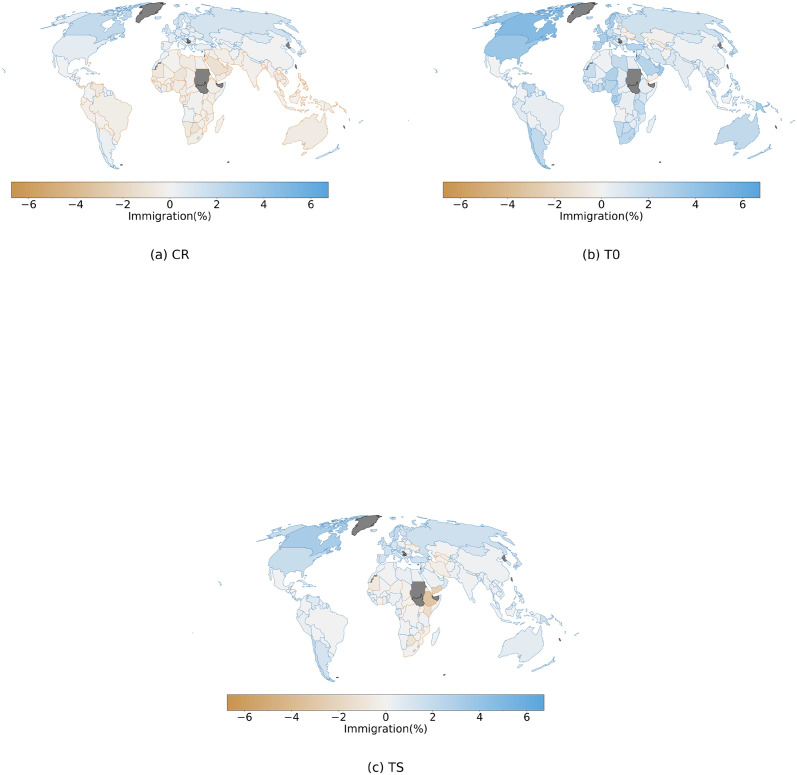
As in Fig 4 but for immigration.

Immigration, under the CR case, shows a similar pattern of impact as observed for emigration but with a larger number of countries showing a reduced immigration (Fig 5a). Since the only varying factor in the CR assumption is the destination country relative GDPc factor, these results largely resemble the figure of impacts of climate change on the GDPc level (Fig 1). Escaping from this pattern, in countries like Mexico, despite a strong negative impact on GDPc, immigration is projected to increase. Importantly, in our model we account separately for the return migration flows (Eq 6b). These flows, not depending directly on economic factors, may play a fundamental role in bilateral migration channels characterized by a large return migration flow (e.g. USA to MEX), and drive results that diverge from common global patterns. Under the T0 assumption we find that immigration increases in almost all the countries and reaches higher levels of impact (Fig 5b). Importantly, we find that many countries see, under the T0 assumption, an opposite impact on immigration compared to the CR case. This highlights the fact that the contribution from the GDPc at the origin dominates over the destination country GDPc factor, not only in defining the patterns of change in emigration but also for those regarding immigration. Under the TS assumption, results are similar as for T0, even though the levels of impact on immigration are smaller (Fig 5c). Altogether, for both the assumptions that include a migration transition mechanism, i.e. T0 and TS, we find a global pattern of higher mobility compared to the CS case. Many countries see an increase in immigration despite the projection of large negative and asymmetric economic impacts, which would reduce their relative capacity to attract migrants. This means that migrants from countries that are impacted negatively may still choose to migrate to another country which also faces negative impacts. This illustrates a more complex mechanism than would be expected by a simplistic implication where “environmental migrants” would move from countries that see damages from climate change to countries that benefit.

**Fig 6:**
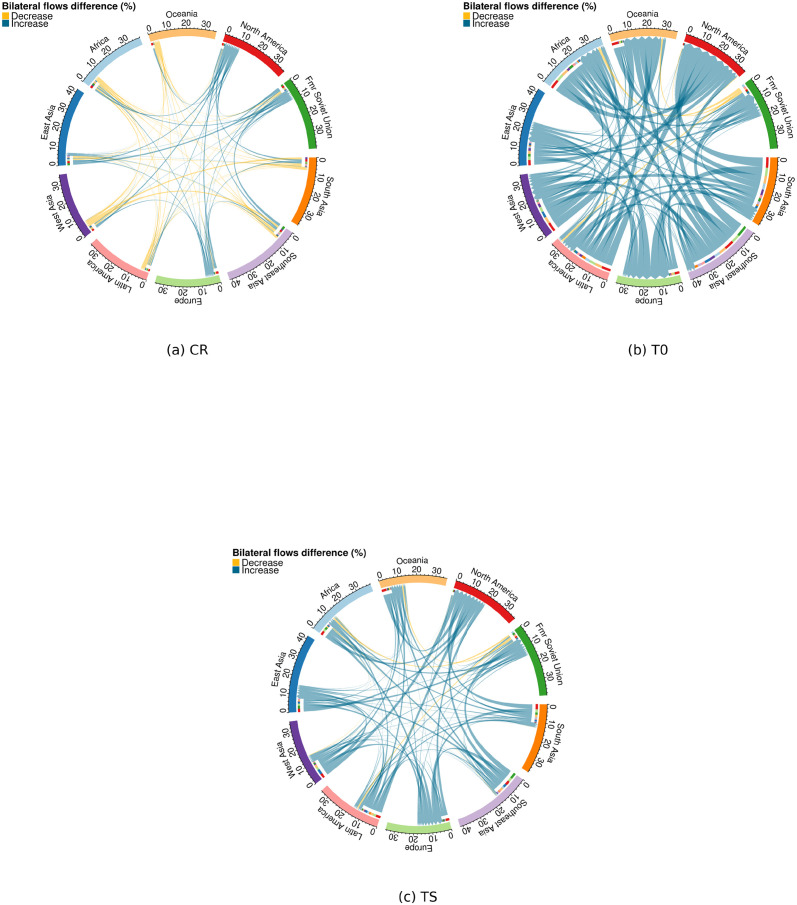
Impact of climate change on bilateral migration flows between ten major world regions under SSP5–8.5 scenario, short-term impact method and the three assumptions regarding emigration rates.

Finally, complementing the results from the country level we also look at the bilateral flows between ten major world regions (Fig 6. We construct these figures by selecting the subset of bilateral flows between countries included in each region (S2 Table in S1 File). We sum up the country-level bilateral flows for each region for both the baseline case and climate change scenario. We then calculate the difference between them, for each bilateral flow between regions. Under the CR assumption, we find that climate change increases migration to North America, Europe, East Asia, and the Former Soviet Union, from most or all other world regions (Fig 6a). All other between-region flows see a decrease due to climate change. In line with the global-level results discussed above, the climate change effect on migration is overall relatively small under this assumption.

Under the T0 assumption, we find much larger effects, mostly increases, in both migration between and within regions and within them (Fig 6b). These increases generally amplify existing migration routes, i.e. we find increases in migration from poorer to richer regions of the world. However, we also find increases e.g. in migration from different parts of Asia to Africa. Note that these effects are expressed as percentage of the baseline flow size, so that large relative changes also in small flows become prominently visible. Thus, these figures are indicative of the relative effect of climate change on between-country flows, but do not show which regions are more affected than others in absolute terms. When the climate change effect is instead expressed in absolute terms, it becomes visible that changes in flows to poor regions like Africa are almost completely due to internal migration, which increases substantially in all regions (S5 Fig in S1 File).

When considering the TS assumption we find very similar results as in T0 but with values reaching lower levels of relative difference (Fig 6c). Assumptions T0 and TS, compared to the CR case, producing projections of increasing regional bilateral migration flows show that, under the migration transition assumption, economic constraints at the origin country are a major factor inhibiting global migration movements and in general driving the patterns of both emigration and immigration response to economic climate change impacts. Values, in percentage, show the difference between the climate change case and the baseline in relative terms of the baseline case. Increases represent cases where migration under the climate change impact scenario is larger than in the baseline scenario. The external thicker arc defines the region of origin while the smaller internal arc shows the region of destination. Arrows point to the destination region. Flows represented are mean values for the period of 3°C global warming and averaged along the climate models dimension.

## 4 Discussion

Given the severe and widespread impacts of climate change, and its potential to alter patterns of economic productivity and development, knock-on effects on international migration appear likely. In particular, if countries undergo a migration transition along with rising productivity, then productivity losses (or gains) due to climate change would slow down (or speed up) this transition, and thus directly interfere with the long-term development of migration patterns. However, with current models and data this hypothesis can neither be validated nor invalidated with certainty. Against this backdrop, our study was meant to explore the range of potential outcomes. Using the most recent models for both, the dependence of global migration on national incomes, and the impact of climate change on national incomes, and for a global warming level of 3°C above pre-industrial, we have demonstrated the effects of climate change on migration through this particular macroeconomic channel, depending on what assumptions are made about the migration transition.

We find that even when migration rates are assumed constant (CR), climate change may act to significantly increase some migration flows, and decrease others, by reorganizing the relative attractiveness of destination countries. These changes are not visible in the net level of mobility, i.e. the global total number of moves, because increases and decreases cancel each other out; highlighting the importance of looking beyond global net effects when studying migration under climate change. Climate change impacts become much larger when countries are assumed to undergo a long-term migration transition along the emigration-GDPc relationship estimated from historical data (T0). In this case, the global total number of moves per 5-year period is projected to increase, by between half a percent and three percent depending on the climate model and socio-economic development scenario. Again, this global number partly hides the full effect of climate change because some flows get increased while others get decreased. Finally, assuming that the emigration-GDPc relation is dynamic and shifts along with global average income (TS) leads to results that are in between the CR and T0 assumptions; again affecting migration flows by several percentage points for many climate models and scenarios.

Our results rely on two different methods for calculating economic effects from climate change impacts [13]. Expanding on previous approaches (e.g. [12, 39]), these methods are thought to improve the analysis on both spatial and temporal scales. Indeed, on the spatial scale they use subnational GDPc data, abandoning the long standing approach using national level GDPc. On the temporal scale they disentangle weather variability impacts (annual scale) from climate impacts (decades). Despite the improvements, these methods follow the majority of recent studies (e.g. [40–45]), focusing only on the temperature variable and its effects on the economic activity. Recently it has been shown that this channel is not the only one through which climate impacts the economy [46]. Therefore our estimates, using two different methods and one climate variable to calculate the GDPc impacts, give an indication of the possible range of outcomes, but not a complete one.

The migration model, on the other hand, includes several important features that set it apart from previous studies [47–51]: (i) it considers bilateral flows between more than 170 countries globally, resulting in an unprecedented spatial coverage, (ii) it considers separately return and transit migration flows, augmenting the resolution on the type of bilateral migration flows with respect to previous works, (iii) it represents the empirically observed non-linear relationship between origin-country incomes and emigration rates, through the “migration hump” function.

Nonetheless, our modeling approach also involves important simplifications and limitations. For instance, our projections of baseline population distribution, broken down by place of birth, rely on country-level natural population change rates applied to all migrant stocks uniformly. They ignore the effects of transient migration that would alter the migrant stock distribution between now and the future time period analyzed; effects that would need to be taken into account for a quantitative prediction exercise. However, for our present analysis, we consider this simplification useful because it facilitates comparison between future and present migration patterns, both assuming the same initial distribution of relative migrant stocks. The same goes, importantly, for feedbacks from altered migration patterns on economic development and GDP [8], which we ignore for the sake of simplicity and transparency in our analysis of climate change effects. It is important to stress out that our modeling study focuses only on international migration. Internal, i.e. within-country, migration is an important phenomenon that would likely face impacts due to future climate change. Our global approach, and country-level population and GDPc data do not allow for carrying out the same study at a higher spatial resolution.

International migration models often include dyadic variables such as geographical distance, colonial links, or common language, between origin and destination country. However, these can become insignificant once bilateral migrant networks are explicitly accounted for by including a diaspora variable ([52], p. 508). This is also the case for the present model specification, where these dyadic variables were estimated to be insignificant, and therefore neglected [26]. Moreover, the geographical distance in particular shows up more significantly in log-transformed models, while untransformed models such as ours predict more limited effects of distance [53, 54].

We acknowledge that international migration might be driven also by other macroeconomic factors as wage differentials and employment rates. We have opted for using only the GDPc because of the global coverage of GDPc projections data and the fact that we want to maintain the model parsimonious in the number of covariates. Moreover, by including the migration “hump function” we manage to capture a more complex dynamics of migration than that suggested by neoclassical economic theory [55] which would project decreasing migration flows as the wage differential decreases [8]. Indeed, observed migration flows have shown a more complex dynamics, with emigration rates from developing countries increasing during a first stage of economic development [15, 56].

The model also does not account for differences in within-country income distributions, nor for changes in the shape of these distributions; i.e. changes in within-country inequality. These distributions clearly differ between countries, and how they will change is likely going to depend on climate change impacts too. Another important effect comes from immigration policies, which affect not only immigration into high income countries [36], but also return migration from these countries [37]. In our model, they are lumped together with other unobserved, country-specific variables in country-specific scaling factors. Implementing these different dimensions of variation will be an important step in our future works for improving our analysis.

Despite these limitations, our results for the first time provide a transparent and nuanced analysis of the potential impacts of future climate change on international migration via one specific channel: the impact on national economic productivity. Our results should be interpreted as an exploratory step towards a more comprehensive and mechanistic approach of quantifying the impact of climate change on international migration, that should serve to inform future model development and data analysis in the field. Further research is needed for improving the understanding and modeling of the mechanisms driving international migration, and the related pathways of climate change impacts on migration.

## Supporting information

S1 File Collection of data, tables and figures used as supporting information. (PDF)
