# Supplementary material for: Climate change and international migration: Exploring the macroeconomic channel
Source: PLoS One. 2022 Nov 16;17(11):e0276764. doi: 10.1371/journal.pone.0276764 (PMC9668176; doi:10.1371/journal.pone.0276764)
Supplement: S1 File — (PDF) [file pone.0276764.s001.pdf]

Climate change and international migration: Exploring the macroeconomic channel

September 16, 2022

1 Supplementary Data

When temperature data was missing we have used the temperature of the nearest country. All these cases appear for small countries. In the following we report these countries and their substitute, using the 3digits ISO code, in the format "country with missing data: substitute country": BHR: SAU, BRB: TTO, HKG: CHN, LCA: TTO, MAC:CHN, MLT: TUN, PSE: ISR, SGP: MYS, TLS: IDN, TON: FJI, VUT: FJI.

List of countries and years for which GDPc values were missing and have been extrapolated from the past: SOM(years 2010,2015), ERI(year 2015).

Table S1: Year defining the 30-years period of $3°C$ global warming reached by each GCM and SSP scenario. The period of 30-years is defined as $[t-14, t+15]$, where t is the reported year.

Climate model	SSP3-7.0	SSP5-8.5
IPSL-CM6A-LR	2060	2055
EC-Earth3	2055	2050
MPI-ESM1-2-HR	2080	2070
UKESM1-0-LL	2045	2045
CNRM-CM6-1	2065	2060
GFDL-ESM4	2070	2065
MRI-ESM2-0	2065	2060
CanESM5	2050	2045
CNRM-ESM2-1	2070	2060
MIROC6	2075	2065

Table S2: Estimates of the country-specific scaling factors as described in the main paper (Model estimation). The values are rounded off to three decimals digit. The countries are grouped by region. These regions correspond to the those used throughout the paper.

Country	$\tilde{a}_j$	$\tilde{b}_i$	Country	$\tilde{a}_j$	$\tilde{b}_i$
Region: Africa					
AGO	0.000	1.315	LSO	4.268	0.000
BDI	0.000	3.053	MAR	0.000	7.456
BEN	2.008	0.679	MDG	1.287	4.836
BFA	0.264	2.171	MLI	0.295	2.599
BWA	2.621	0.759	MOZ	0.682	0.642
CAF	1.745	17.820	MRT	1.096	0.871
CIV	1.884	1.619	MUS	1.207	0.770
CMR	2.424	1.251	MWI	0.222	4.512
COD	0.531	3.877	NAM	0.848	0.872
COG	2.578	1.079	NER	4.301	2.136
COM	0.000	2.133	NGA	1.934	1.055
CPV	0.626	3.365	RWA	0.808	5.498
DJI	2.907	0.942	SEN	1.266	1.455
DZA	0.804	0.746	SLE	1.173	6.518
EGY	1.458	0.844	SOM	0.000	9.477
ERI	1.674	5.035	STP	0.000	3.402
ETH	5.023	0.893	SWZ	0.527	0.263
GAB	1.738	3.184	TCD	0.495	0.000
GHA	2.641	0.423	TGO	2.781	1.597
GIN	2.363	5.052	TUN	1.304	0.000
GMB	1.916	0.989	TZA	3.577	3.128
GNB	0.555	1.642	UGA	1.319	1.706
GNQ	14.238	0.000	ZAF	4.014	0.624
KEN	3.170	0.896	ZMB	0.480	4.239
LBR	0.000	2.758	ZWE	0.956	2.725
LBY	0.688	0.902			
Region: East Asia					
CHN	0.755	1.061	KOR	1.384	1.228
HKG	0.616	1.160	MAC	0.651	1.084
JPN	0.881	1.076	MNG	1.821	8.111
Region: Europe					
ALB	0.000	23.077	HUN	1.172	0.829

AUT	1.157	1.137	IRL	1.057	0.128
BEL	0.780	1.067	ISL	1.352	1.915
BGR	2.191	0.000	ITA	1.327	1.053
BIH	0.000	21.379	LUX	1.037	1.167
CHE	1.157	1.352	MKD	1.174	0.000
CZE	1.005	1.034	MLT	2.168	1.013
DEU	0.972	1.161	NLD	0.737	1.105
DNK	1.030	1.106	NOR	1.203	0.967
ESP	1.141	1.190	POL	0.482	1.080
FIN	1.071	0.000	PRT	0.575	1.025
FRA	0.795	1.124	ROU	1.390	1.152
GBR	1.233	0.947	SVK	0.988	1.463
GRC	0.000	1.023	SVN	0.850	0.899
HRV	0.310	0.794	SWE	1.011	1.164
Region: Fmr Soviet Union					
ARM	0.768	1.839	LVA	0.445	1.711
AZE	0.265	0.000	MDA	0.645	1.094
BLR	0.634	0.394	RUS	1.062	0.788
EST	0.461	1.409	TJK	0.611	2.695
GEO	0.000	14.845	TKM	0.526	1.837
KAZ	0.593	1.667	UKR	0.710	0.453
KGZ	0.661	2.190	UZB	0.741	0.830
LTU	0.432	2.576			
Region: Latin America					
ARG	0.848	1.135	HTI	0.000	44.716
BHS	1.643	1.011	JAM	0.000	0.000
BLZ	1.188	0.825	LCA	1.291	0.000
BOL	0.393	0.414	MEX	0.000	0.000
BRA	1.006	0.846	NIC	0.000	8.118
BRB	0.521	0.148	PAN	1.268	0.999
CHL	3.144	1.132	PER	4.553	35.075
COL	0.000	0.338	PRI	0.000	2.422
CRI	1.432	0.965	PRY	0.531	1.757
CUB	0.000	3.857	SLV	0.000	25.262
DOM	1.739	1.003	SUR	0.000	0.106
ECU	1.309	0.955	TTO	0.173	0.000
GTM	0.000	1.153	URY	0.512	2.788
GUY	0.000	11.733	VCT	0.927	4.387
HND	0.000	0.609	VEN	1.078	4.798

Region: North America					
CAN	1.152	1.014	USA	1.062	1.034
Region: Oceania					
AUS	1.091	1.074	SLB	0.641	10.399
FJI	0.000	7.908	TON	0.000	2.630
NZL	1.111	1.272	VUT	0.667	2.860
PNG	1.492	2.212	WSM	0.000	9.344
Region: South Asia					
AFG	0.000	3.034	LKA	0.197	2.245
BGD	2.180	2.826	MDV	3.248	0.697
BTN	0.622	0.950	NPL	0.974	4.400
IND	1.408	0.867	PAK	1.156	1.659
IRN	0.896	1.484			
Region: Southeast Asia					
AFG	0.000	3.034	LKA	0.197	2.245
BGD	2.180	2.826	MDV	3.248	0.697
BTN	0.622	0.950	NPL	0.974	4.400
IND	1.408	0.867	PAK	1.156	1.659
IRN	0.896	1.484			
Region: West Asia					
ARE	0.864	1.083	OMN	1.405	1.076
BHR	1.096	1.011	PSE	0.000	0.930
CYP	1.737	0.000	QAT	0.893	1.049
IRQ	0.808	0.353	SAU	0.912	1.117
ISR	0.855	1.286	SYR	1.044	3.884
JOR	1.323	0.649	TUR	2.654	0.682
KWT	0.866	1.154	YEM	6.035	1.157
LBN	0.784	0.619			

Table S3: **Estimates for different specification of the migration model, including dyadic variables.**

Variable	*Parameter*	Model 1	2	3	4	5
Emigration and transit migration						
Intercept	a	0.233 ± 0.004	0.075 ± 0.009	0.08 ± 0.01	0.08 ± 0.01	0.07 ± 0.01
Diaspora	α_p	0.943 ± 0.003	0.951 ± 0.003	0.953 ± 0.004	0.954 ± 0.004	0.978 ± 0.004
Dest. GDP	α_g	0.19 ± 0.01	0.17 ± 0.01	0.17 ± 0.01	0.17 ± 0.01	0.10 ± 0.02
log(Distance)	α_d	-	0.57 ± 0.06	0.53 ± 0.07	0.53 ± 0.07	0.70 ± 0.07
Border	α_b	-	-	-0.02 ± 0.02	-0.02 ± 0.02	-0.07 ± 0.02
Common official language	α_c	-	-	-	0.01 ± 0.01	0.07 ± 0.01
Colonial ties	α_l	-	-	-	-	-0.28 ± 0.02
Orig. GDP	γ	-0.0016	-0.0016	-0.0016	-0.0016	-0.0016
	$\widehat{G}$	$ 35301	35301	35301	35301	35301
	$\widetilde{G}$	$ 929	929	929	929	929
Return migration						
Intercept	b	0.124 ± 0.001	0.35 ± 0.06	0.35 ± 0.06	0.35 ± 0.06	0.35 ± 0.06
log(Distance)	α_{d_r}	-	-0.51 ± 0.09	-0.51 ± 0.09	-0.51 ± 0.09	-0.049 ± 0.09
	R^2	0.69	0.69	0.69	0.69	0.70

Estimated values, through a NLS method, for the global parameters of different specifications of the migration model, reported with a confidence level of 99%. In this estimation the parameters of the origin GDP variable are not estimated but set to the reported values coming from the first step estimation.

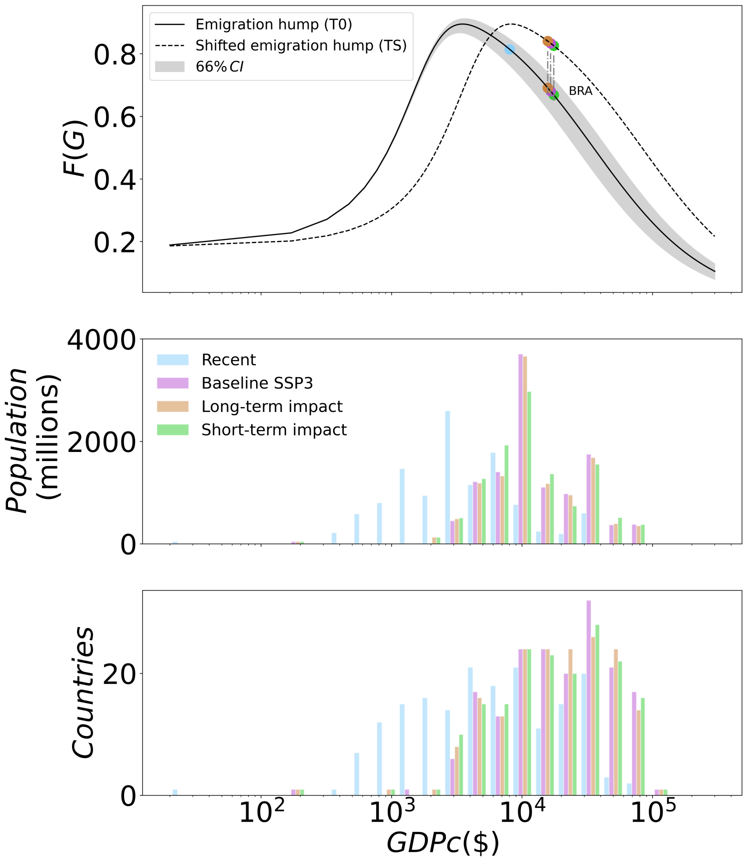

Figure S1: As in Fig. 1 in the main paper but for SSP3-7.0.

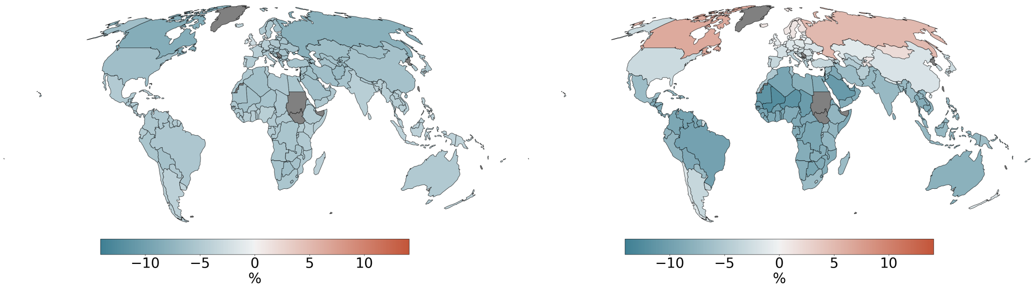

(a) (b)

Figure S2: As in Fig. 2 of the main paper but for SSP3-7.0.

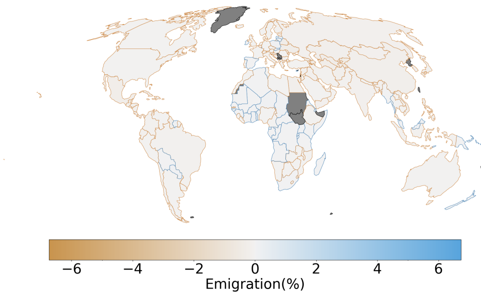

(a) CR

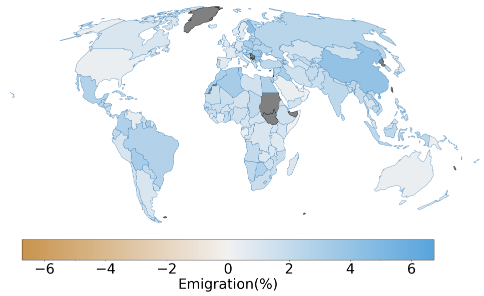

(b) T0

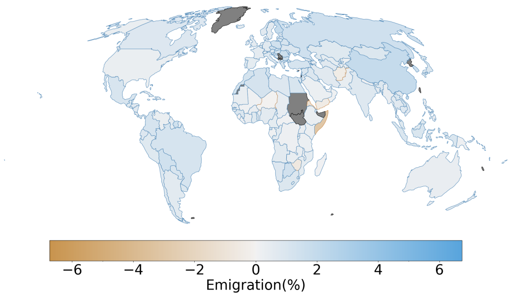

(c) TS

Figure S3: As in Fig. 4 of the main paper but for the long-term climate change impact.

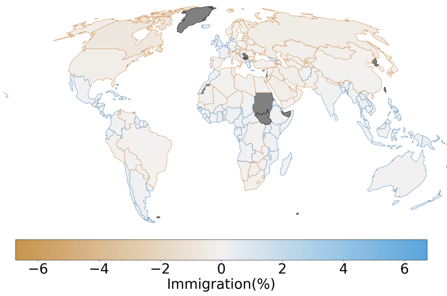

(a) CR

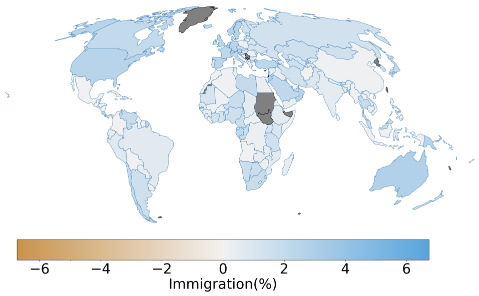

(b) T0

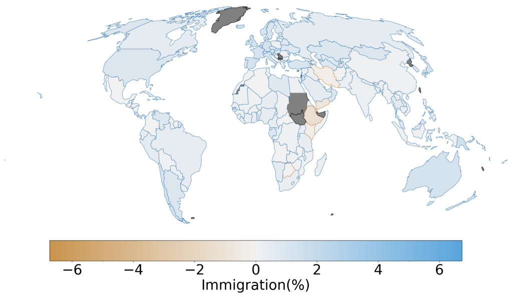

(c) TS

Figure S4: As in Fig. 5 of the main paper but for the long-term climate change impact.

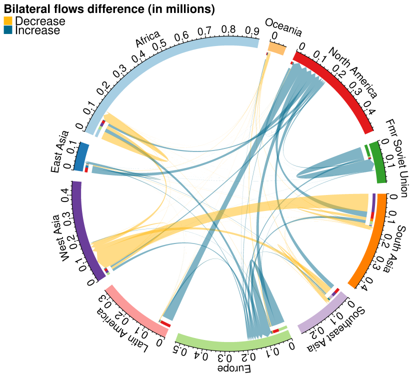

(a) CR

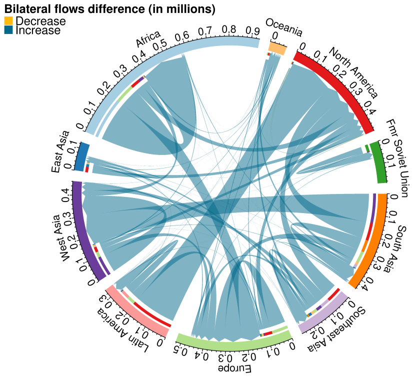

(b) T0

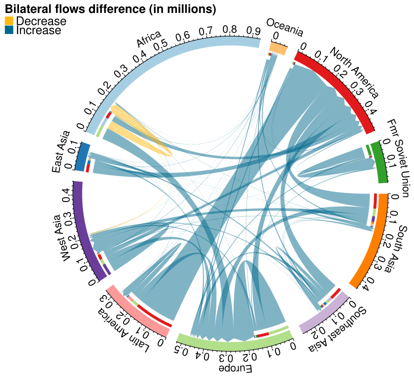

(c) TS

Figure S5: As in Fig. 6 in the main paper but for the difference in absolute terms.

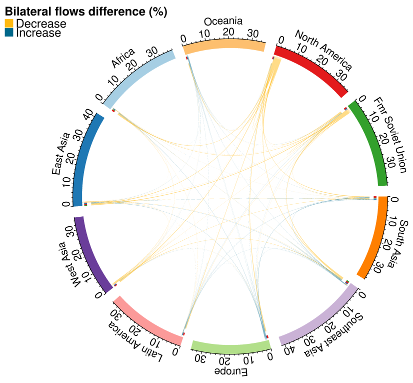

(a) CR

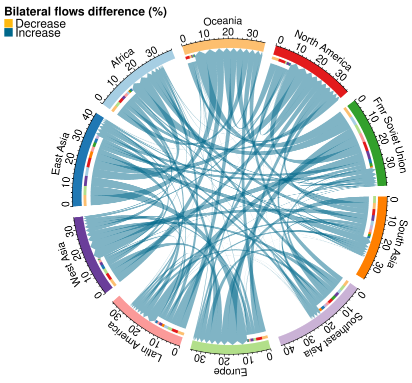

(b) T0

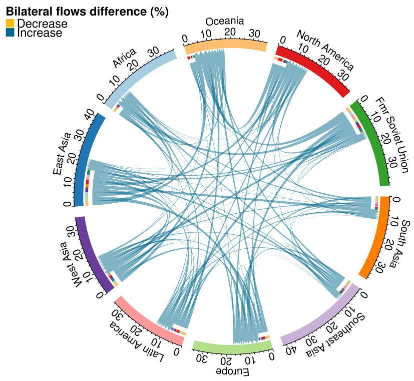

(c) TS

Figure S6: As in Fig. 6 in the main paper but for the long-term impact method.

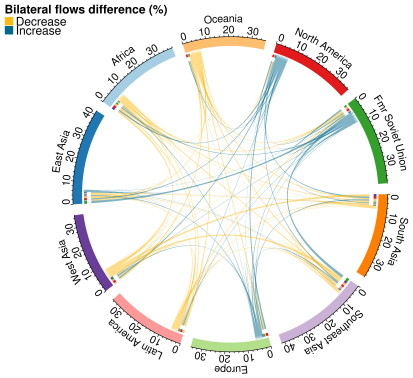

(a) CR

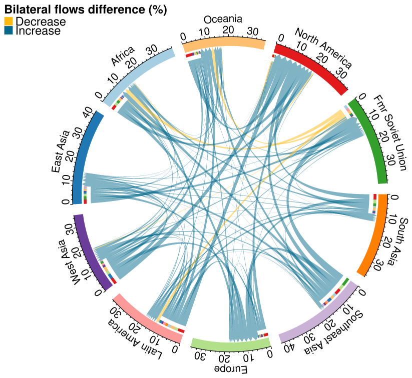

(b) T0

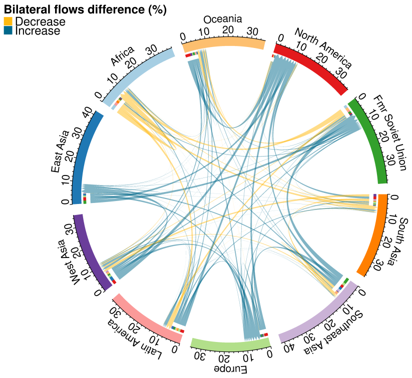

(c) TS

Figure S7: As in Fig 6 in the main paper but for SSP3-7.0.

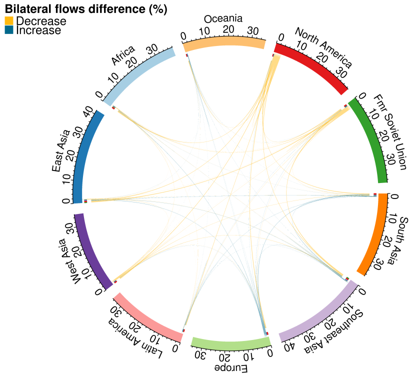

(a) CR

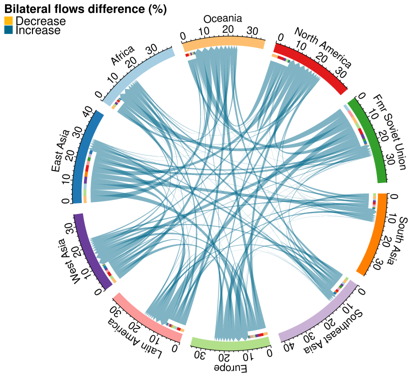

(b) T0

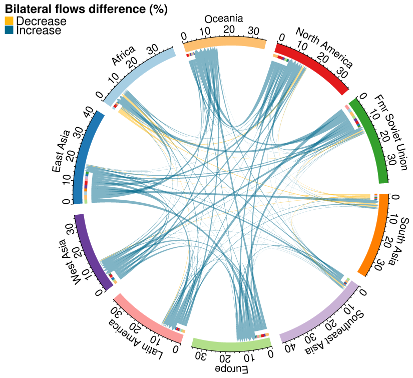

(c) TS

Figure S8: As in Fig. 6 in the main paper but for SSP3-7.0 and long-term impact method.

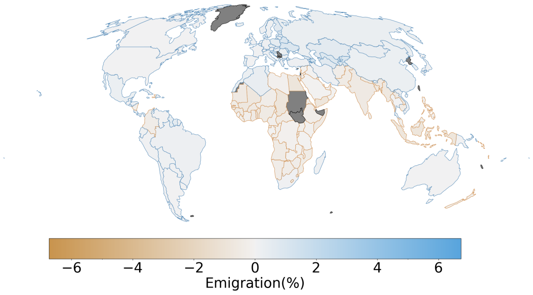

(a) CR

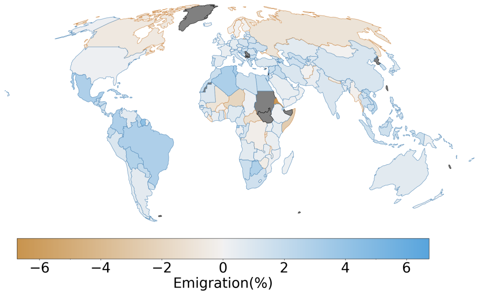

(b) T0

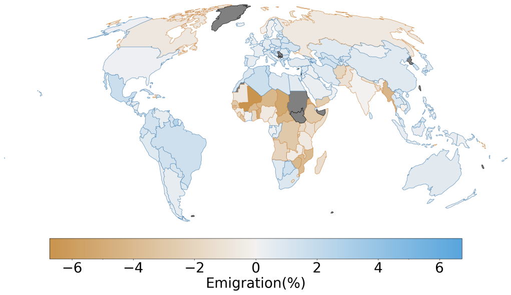

(c) TS

Figure S9: As in Fig. 4 of the main paper but for the SSP3-7.0.

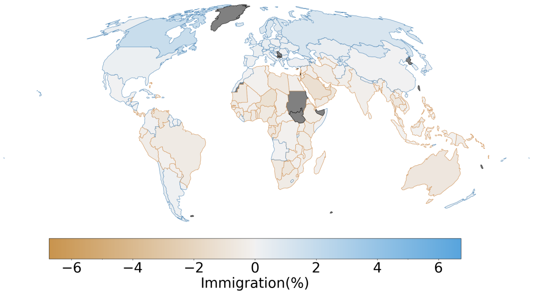

(a) CR

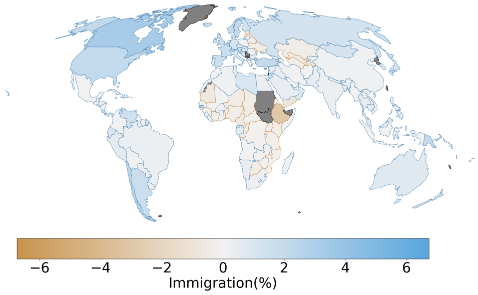

(b) T0

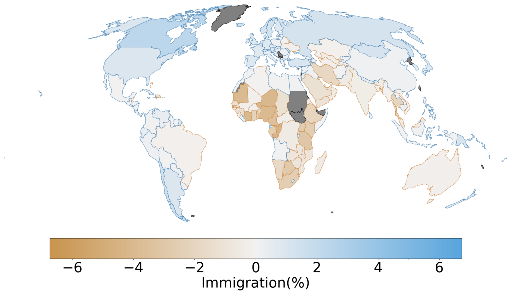

(c) TS

Figure S10: As in Fig. 5 of the main paper but for SSP3-7.0.

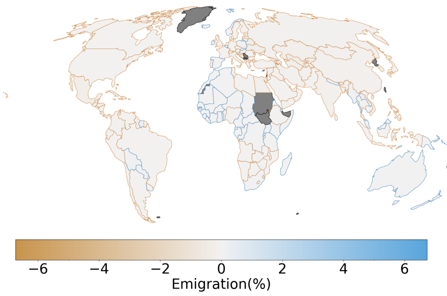

(a) CR

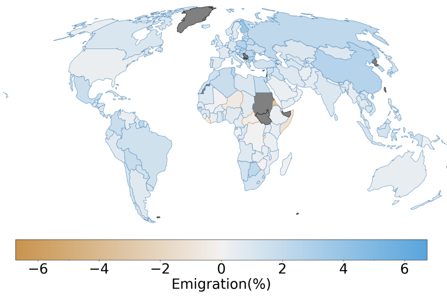

(b) T0

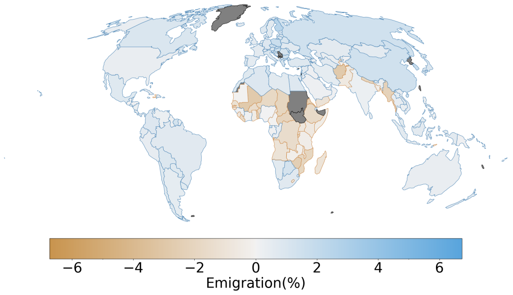

(c) TS

Figure S11: As in Fig. 4 of the main paper but for the long-term climate change impact and SSP3-7.0.

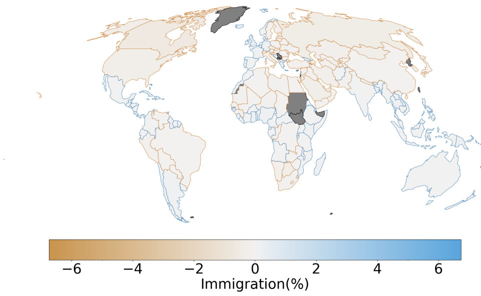

(a) CR

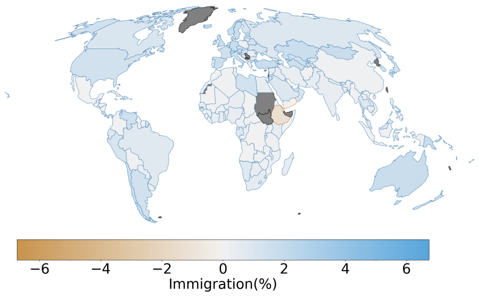

(b) T0

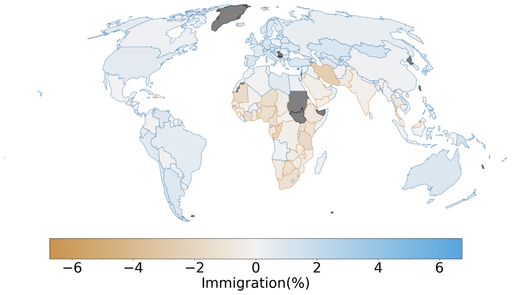

(c) TS

Figure S12: As in Fig. 5 of the main paper but for the long-term climate change impact and SSP3-7.0.
